# Recurrent glioblastoma metastatic to the lumbar vertebra: A case report and literature review: Surgical oncology

**DOI:** 10.3389/fonc.2023.1101552

**Published:** 2023-02-16

**Authors:** Ako Matsuhashi, Shota Tanaka, Hirokazu Takami, Masashi Nomura, Masako Ikemura, Yoshitaka Matsubayashi, Yusuke Shinoda, Keisuke Yamada, Yu Sakai, Yasuaki Karasawa, Shunsaku Takayanagi, Nobuhito Saito

**Affiliations:** ^1^ Department of Neurosurgery, The University of Tokyo Hospital, Tokyo, Japan; ^2^ Department of Pathology, The University of Tokyo Hospital, Tokyo, Japan; ^3^ Department of Orthopedic Surgery, The University of Tokyo Hospital, Tokyo, Japan; ^4^ Department of Rehabilitation Medicine, Saitama Medical University Hospital, Saitama, Japan

**Keywords:** glioblastoma, vertebral metastasis, craniotomy, methylation array analysis, copy number alteration, chromosomal instability

## Abstract

**Background:**

Glioblastoma is a malignant tumor, and its prognosis is as poor as 1.5 to 2 years. Most cases recur within one year even under the standard treatment. The majority of recurrences are local, and in rare cases, metastasize mostly within the centra nervous system. Extradural metastasis of glioma is exceedingly rare. Here, we present a case of vertebral metastasis of glioblastoma.

**Case presentation:**

We present a 21-year-old man post total resection of the right parietal glioblastoma, diagnosed with lumbar metastasis. He originally presented with impaired consciousness and left hemiplegia and underwent gross total resection of the tumor. Given the diagnosis of glioblastoma, he was treated with radiotherapy combined with concurrent and adjuvant temozolomide. Six months after tumor resection, the patient presented with severe back pain, and was diagnosed as metastatic glioblastoma on the first lumbar vertebrae. Posterior decompression with fixation and postoperative radiotherapy were conducted. He went on to receive temozolomide and bevacizumab. However, at 3 months after the diagnosis of lumbar metastasis, further disease progression was noted, and his care was transitioned to best supportive care. Comparison on copy number status between primary and metastatic lesions on methylation array analysis revealed more enhanced chromosomal instability including 7p loss, 7q gain and 8 gain in the metastatic lesion.

**Conclusion:**

Based upon the literature review and our case, younger age of initial presentation, multiple surgical interventions, and long overall survival seem to be the risk factors of vertebral metastasis. As the prognosis of glioblastoma improves over time, its vertebral metastasis is seemingly more common. Therefore, extradural metastasis should be kept in mind in the treatment of glioblastoma. Further, detailed genomic analysis on multiple paired specimens is mandated to elucidate the molecular mechanisms of vertebral metastasis.

## 1 Introduction

Glioblastoma is a malignant tumor, classified as grade 4 in World Health Organization (WHO) classification of central nervous system (CNS) tumors 2021, and its prognosis is as poor as 1.5 to 2 years (1, 2). Most cases recur within one year even under the standard treatment of surgical resection, radiation therapy and chemotherapy. The vast majority of recurrences are local, and in rare cases, metastatic mostly within the CNS. Extradural metastasis is considered exceedingly rare due to the presence of blood brain barrier (3). Here, we report a case of vertebral metastasis of glioblastoma.

This study was reported in agreement with principles of the CARE guidelines (4). Written informed consent was obtained from the individual and the patient’s legal guardian for the publication of any potentially identifiable images or data included in this article.

## 2 Case description

### 2.1 Clinical course

**Figure 1:**
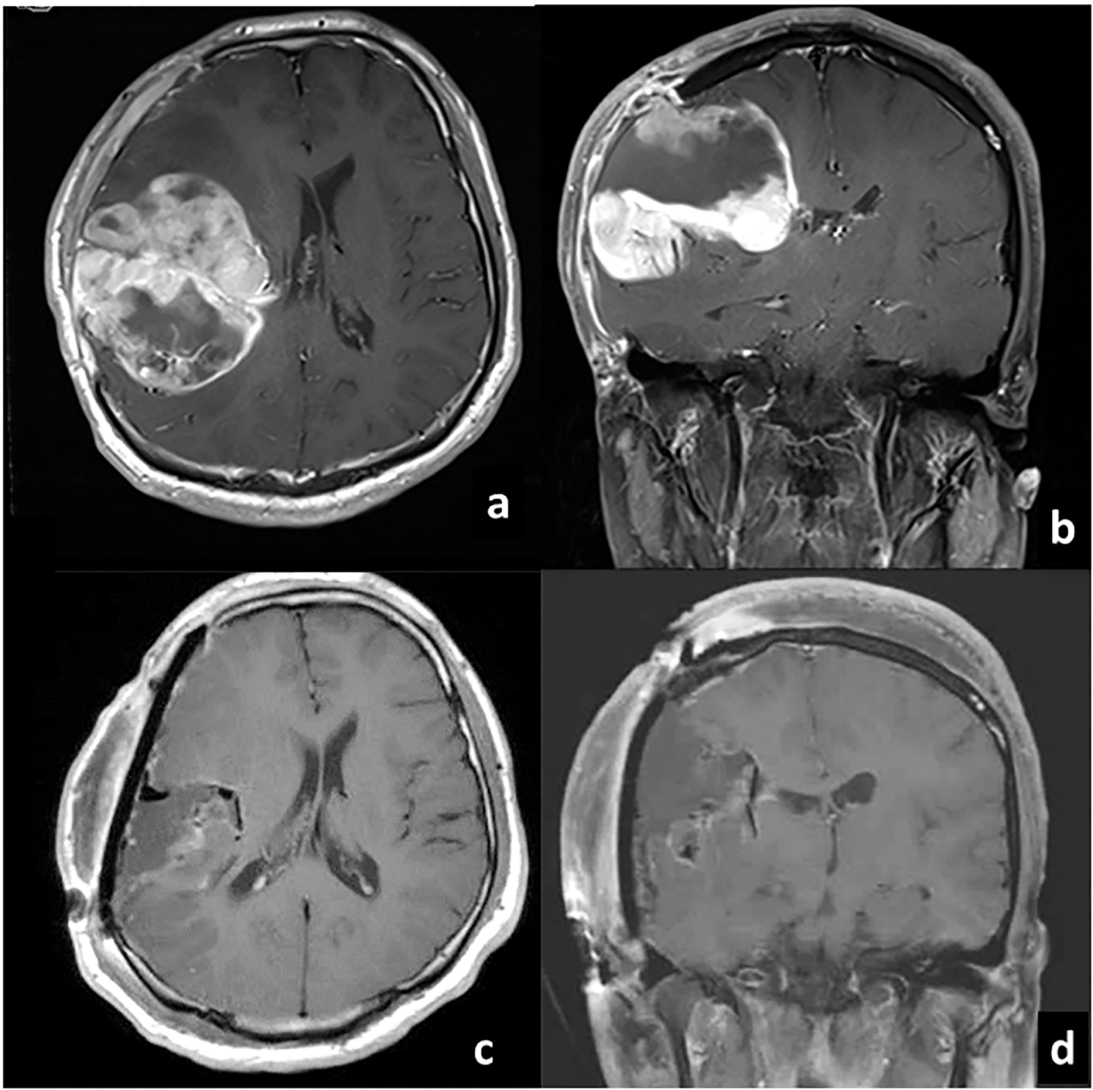
Gadolinium-enhanced T1-weighted MRI before **(A)** axial, **(B)** coronal and after **(C)** axial, **(D)** coronal) gross total resection of glioblastoma at our institute.

A 21-year-old man post total resection of the right parietal glioblastoma was diagnosed with lumber metastasis. At the age of 20, without any significant family history or past medical history, he presented to a local emergency department with impaired consciousness and left hemiplegia. Computed tomography (CT) showed intracranial hemorrhage in the right parietal lobe, and hematoma evacuation was conducted. Dilated veins were observed around the hematoma and the patient was diagnosed with intracranial hemorrhage from venous hemangioma. Five months after the operation, he began to complain of headache, and magnetic resonance imaging (MRI) showed an enhanced lesion in the right hemisphere which was rapidly increasing in size. Partial removal of the mass and external decompression was conducted. Then he was referred to our institute for resection of the remaining mass and adjuvant therapy. Gross total resection of the tumor, placement of carmustine wafer in the resection cavity and cranioplasty were conducted at our institute (**Figure 1**). Pathology was consistent with glioblastoma, isocitrate dehydrogenase (IDH)-wildtype. The patient was ambulatory post-operatively. Radiotherapy combined with concurrent and adjuvant temozolomide (75mg/m^2^/day) per the Stupp regimen was conducted. Intensity Modulated Radiation Therapy (IMRT) of 60Gy was conducted at the edematous area surrounding the tumor, and IMRT of 50Gy at the tumor removal site. Four cycles of temozolomide (150mg/m^2^/day) were administered during the maintenance phase. At the same time, bevacizumab (10mg/kg) was administered twice, two weeks apart.

**Figure 2:**
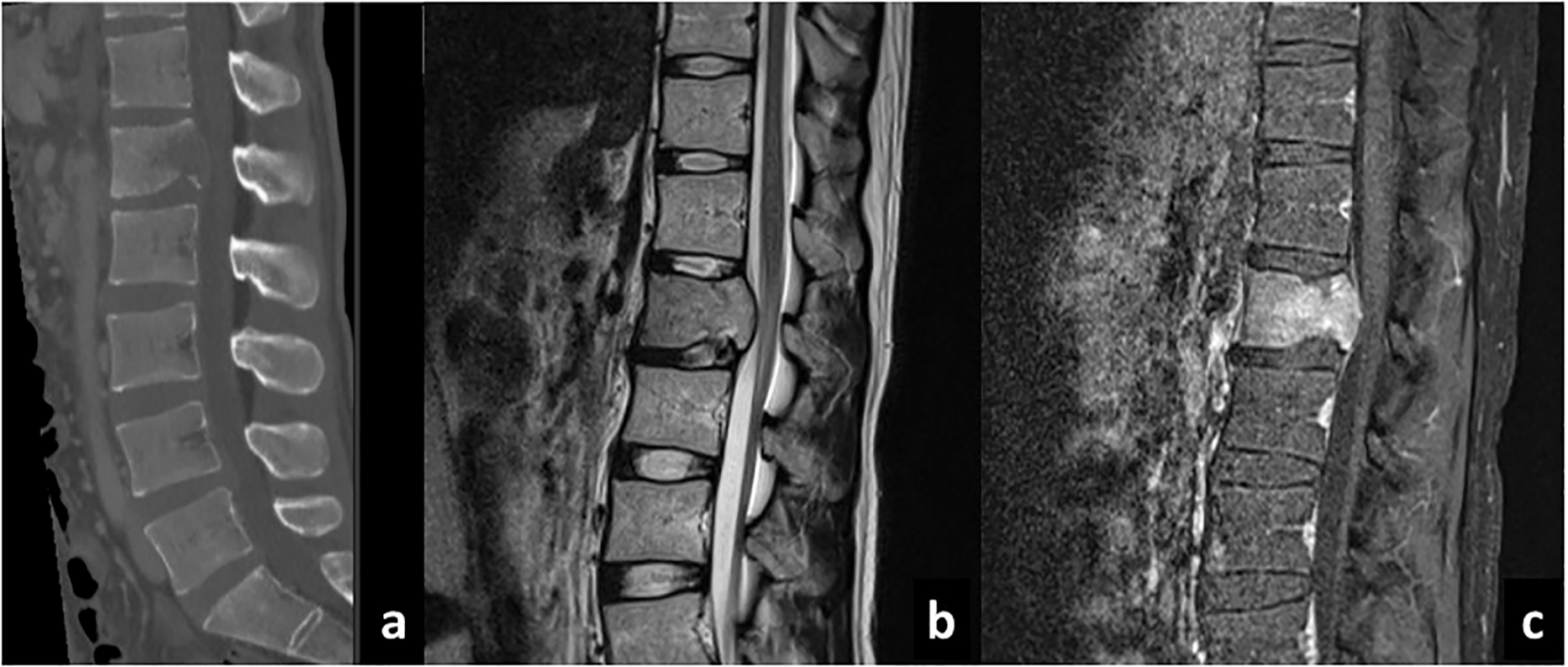
Sagittal CT **(A)**, T2-weighted **(B)**, and gadolinium-enhanced T1-weighted **(C)** MRI sequences of the spine showing development of metastatic lesion.

**Figure 3:**
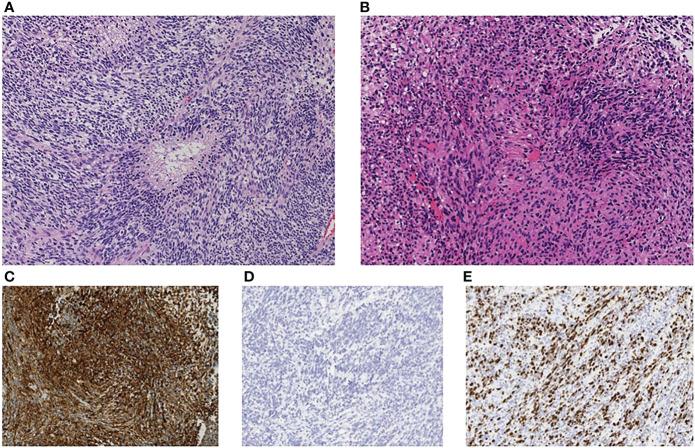
Immunohistopathological investigation showed densely infiltrating spindle-shaped cells with eosinophilic cytoplasm accompanied with regions of necrosis, consistent with glioblastoma **(A)** primary site, **(B)** lumbar metastasis). The cells were positive for glial fibrillary acidic protein (GFAP) **(C)** and negative for mutant isocitrate dehydrogenase 1 (IDH1-R132H) **(D)**. MIB-1 labeling index was 30% **(E)**. The original magnification was x200 **(A–E)**.

At 6 months after the resection of tumor at our institute (14 months after the first operation), the patient presented with severe back pain. CT showed an osteolytic mass on the body of the first lumbar vertebrae and MRI showed an enhanced lesion constricting the spinal canal (**Figure 2**). There was no spinal metastasis. The patient did not present symptoms of spinal cord compression such as bladder and rectal disturbance or lower extremities paralysis. Brain MRI showed no intracranial recurrence, and whole spine MRI and whole-body CT showed no other lesions. Needle biopsy of the mass on the first lumbar vertebrae showed densely infiltrating spindle-shaped cells with eosinophilic cytoplasm accompanied with regions of necrosis, which was consistent with glioblastoma (**Figure 3**). The cells were positive for glial fibrillary acidic protein (GFAP), and its MIB-1 index was 30%. Posterior decompression with fixation and postoperative radiotherapy of 30Gy were conducted to relieve the pain. He further received one more cycle of temozolomide (150mg/m^2^/day) and two more administrations of bevacizumab (10mg/kg, two weeks apart). Three months after the diagnosis of lumbar metastasis, however, disease progression with multiple metastasis to the lymph nodes, the lungs, and the liver was found, prompting the transition to best supportive care.

### 2.2 Epigenetic analysis

This study was approved by the Ethics Committee of the University of Tokyo (#G10028). DNA was extracted from FFPE tissue samples and analyzed using the Illumina Infinium Human Methylation EPIC Bead Chip array according to the manufacturer’s instructions. All DNA methylation analyses were performed using R version 4.1.0. Methylation values were calculated as β values. The following filtering criteria were applied: removal of probes overlapping with single-nucleotide polymorphisms, those mapped to chromosomes X and Y, or the Illumina control probes. Copy number alterations were calculated using signal data from the methylation array using the conumee Bioconductor package version 1.26.0.

**Figure 4:**
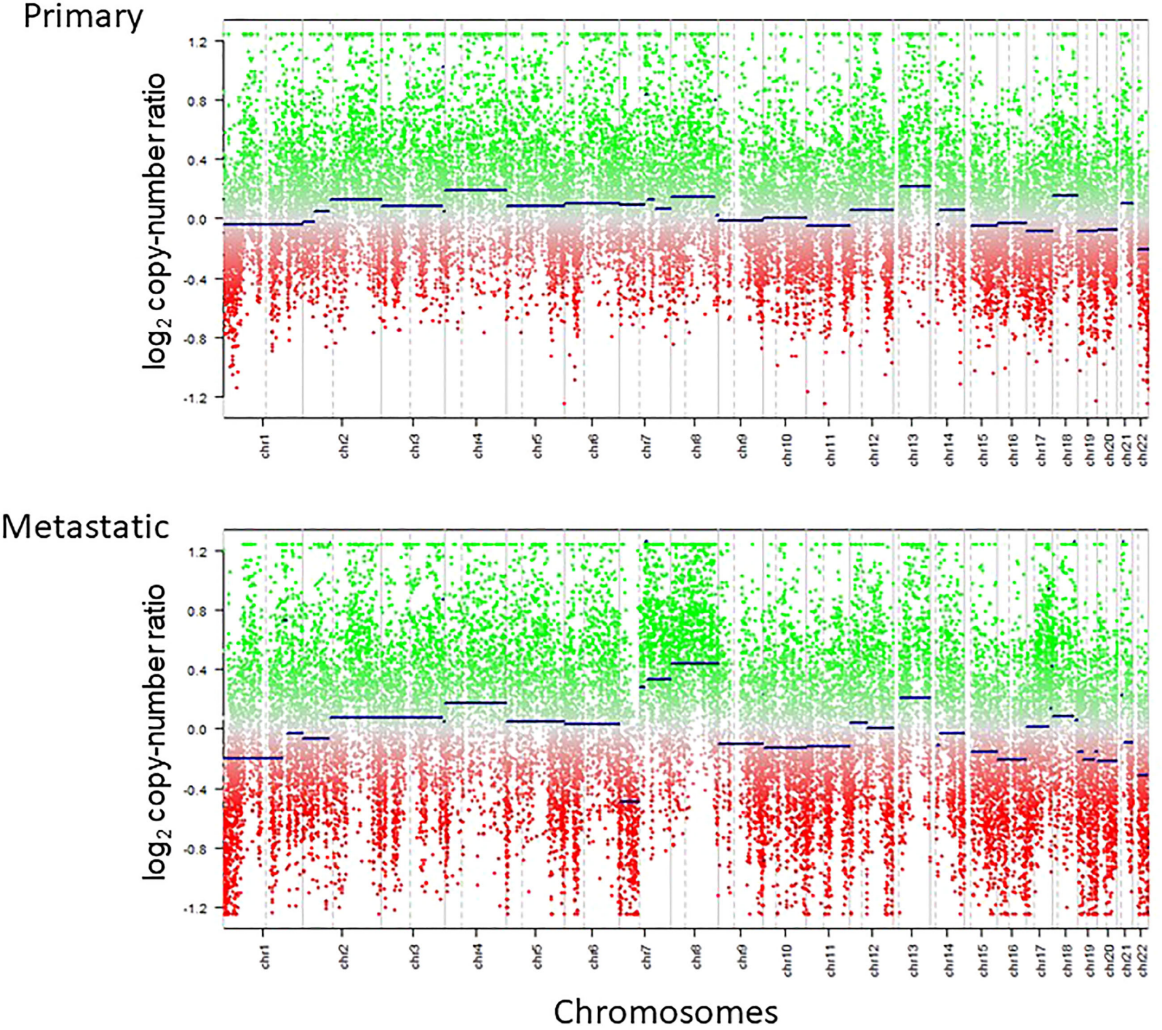
Copy number profiles of primary and metastatic lesions calculated by methylation array are shown. Copy number baseline imbalance was enhanced for the metastatic lesion compared with the primary lesion.

Primary and metastatic samples had overall copy number status in common such as chromosome 1p loss, 16 loss and 22q loss; however, the metastatic sample had more prominent copy number alterations, including newly acquired 4 gain, 7p loss, 7q gain, 8 gain, 13q gain, 19q gain, and 20 loss (**Figure 4**).

## 3 Discussion

In this case illustration, we present an extremely rare case of glioblastoma which metastasized extracranially to the lumbar vertebra during the course of treatment. Glioblastoma was not believed to metastasize outside of the CNS until Davis first reported glioma meningeal metastasis in 1928 (5, 6). Extradural metastasis of glioblastoma is as rare as less than 2% (3). Dense dura around intracranial sinuses preventing tumor cell penetration and lack of a nurturing stroma in other organs to facilitate the survival of glioblastoma cells are some biological obstacles that prevent glioblastoma cells to infiltrate outside the CNS (7). Also, overall low median survival causes patients to die from intracranial hypertension or other complications before extracranial metastasis develop (6).

Vertebral metastasis is exceedingly rare; to the best of our knowledge, there has been 59 cases reported in the literature (**Supplementary Tables 1, 2**) (6, 8–58). Mean age of the 59 patients is 43.9 years (range, 11-70 years), which is much younger than the average age of patients diagnosed with glioblastoma. Moreover, in most cases, glioblastoma metastasis to the vertebral body is accompanied by metastasis to other locations such as the lung, lymph nodes, other bones and the liver. As for the cases with sufficient data, the mean overall survival of the cases is 28.0 months (range, 1-139 months) after the initial diagnosis of glioblastoma, and 8.5 months (range, 0-48 months) after the diagnosis of vertebral metastasis. Overall survival in these cases is longer than the average prognosis of glioblastoma. This suggests that long-surviving glioblastoma patients provide glioblastoma cells adequate time to cause extradural metastasis.

Considering the biological obstacles that prevent glioblastomas from infiltrating outside of the CNS, it can be speculated that deposition of tumor cells into the blood stream or excision of the dura due to surgical interventions may attribute to extracranial metastasis. As for the 59 reported cases, the mean number of surgeries conducted prior to vertebral metastasis was 1.5 (range, 0-5). In our case, the patient first presented with intracranial hemorrhage and the tumor was hypervascular enough to be misdiagnosed for venous hemangioma, allowing tumor cells to be feasibly deposited into the blood stream. Also, multiple surgeries including external decompression were conducted causing defects of the dura and the skull, which could have facilitated the extradural infiltration of glioblastoma cells. Based upon the literature and our case, younger age of initial presentation of glioblastoma and multiple surgical interventions seem to be the risk factors of vertebral metastasis of glioblastoma, though we still lack further statistical evaluation.

Little has been known about the genetic and epigenetic changes seen in the extracranial metastasis of glioblastoma. Copy number alterations have been known to be enhanced at the metastatic site compared with the primary site, in parallel with mutational burden, in systemic cancers such as lung, hepatocellular and urothelial malignancies among others (59–61). This is deemed to primarily reflect the clonal evolution and selection of the tumor cells at the primary site with the gain of metastatic potential (62, 63). Previously, a case of GBM with osseous metastasis was shown to harbor additional copy number alterations plus mutations compared with the intracranial tumor (64). In concordance, our case demonstrated the increased fluctuation of the copy number baseline (**Figure 4**), highly suggestive of the existence of tumor cells with the acquisition of metastatic capability. This is still an under-investigated finding in cases with metastatic glioblastoma and needs to be investigated in a larger cohort of samples.

Due to the improvement in the prognosis of the disease, vertebral metastasis is suspected to be encountered more commonly. Therefore, extradural metastasis of glioblastoma must be included in differential diagnoses in treating patients with glioblastoma. Further studies with detailed genomic analysis on multiple paired tumor specimens are warranted to unravel the molecular mechanisms of vertebral metastasis.

## Data availability statement

The data analyzed in this study is subject to the following licenses/restrictions: This is a case report. All the information is stored in the medical chart. Requests to access these datasets should be directed to STan, stanaka@m.u-tokyo.ac.jp.

## Ethics statement

The studies involving human participants were reviewed and approved by The University of Tokyo. The patients/participants provided their written informed consent to participate in this study. Written informed consent was obtained from the individual and the patient’s legal guardian for the publication of any potentially identifiable images or data included in this article.

## Author contributions

AM and STan contributed to conception and design of the study. AM organized the database. AM and HT performed the statistical analysis. AM and STan wrote the first draft of the manuscript. HT wrote sections of the manuscript. All authors contributed to manuscript revision, read, and approved the submitted version.
