# Supplementary material for: Recurrent glioblastoma metastatic to the lumbar vertebra: A case report and literature review: Surgical oncology
Source: Front Oncol. 2023 Feb 16;13:1101552. doi: 10.3389/fonc.2023.1101552 (PMC9978767; doi:10.3389/fonc.2023.1101552)
Supplement: Supplementary file 1 [file Table_1.docx]

Supplementary Material

# Supplementary Tables

**Supplementary Table 1.** Demographics and outcomes of patients with vertebral metastasis of glioblastoma

| **Characteristics** | **Number of patients (N=59)** |
| --- | --- |
| Sex, male, n | 44 (75%) |
| Age at diagnosis (years) | 43.9 (range, 11-70) |
| Location, n |  |
| Cervical | 7 (11%) |
| Thoracic | 18 (31%) |
| Lumber | 13 (22%) |
| Multilevel | 5 (8.5%) |
| Unknown | 16 (27%) |
| Treatment of primary glioblastoma |  |
| No surgery | 2 (3.4%) |
| Number of surgeries prior to metastasis | 1.5 (range 0-5) |
| Mean survival after initial diagnosis (months) | 28.0 (range, 1-139) |
| Mean survival after vertebral metastasis (months) | 8.5 (range, 0-48) |

**Supplementary Table 2.** Summary of patients with vertebral metastasis of glioblastoma

| Authors, year | Age (years) /sex | Spinal level | Number of craniotomies prior to metastasis | Survival after primary glioblastoma diagnosis (months) | Survival after vertebral metastasis diagnosis (months) |
| --- | --- | --- | --- | --- | --- |
| Winkelman, 1952 | 52/M | T4 | 2 | 16 | N/A |
| Wisiol, 1962 | 31/M | T6 | 1 | 11 | 0.83 |
| Nigogosyan, 1962 | 40/M | N/A | 2 | 14 | N/A |
| Smith, 1969 | 36/M | N/A | 2 | 12 | N/A |
| Smith, 1969 | 63/F | N/A | 1 | 11 | N/A |
| Smith, 1969 | 36/M | T10, 11 | 1 | 14 | N/A |
| Anzil, 1970 | 53/F | T11 | 0 | 12 | N/A |
| Takeda, 1971 | 11/M | T12 | 3* | 96 | N/A |
| Cooper, 1974 | 59/F | C,T,L | 1 | 6 | N/A |
| Cooper, 1974 | 24/M | N/A | 1 | 8 | N/A |
| Hulbanni, 1976 | 63/M | L | 0 | 1 | N/A |
| Schatzki, 1977 | 56/M | T, L | 2 | 96 | 6 |
| Slowik, 1980 | 43/M | T5 | 2 | 24 | N/A |
| Slowik, 1980 | 24/M | T3, 5, L3 | 1 | 16 | N/A |
| Dietz, 1981 | 24/M | T | 1 | 6 | N/A |
| Sadik, 1984 | 48/M | L4 | 1 | N/A | N/A |
| Friedman, 1986 | 52/M | N/A | 1 | 32 | 3 |
| Haddon, 1989 | 31/M | T10-11 | 2 | 8 | 1 |
| Lampl, 1990 | 32/M | L4 | 1 | 10 | 1 |
| Myers, 1990 | 11/F | L | 0** | N/A | N/A |
| Chesnut, 1993 | 42/M | C6, 7 | 1 | 23 | 14 |
| Mihara, 1994 | 35/F | T1 | 2 | 14 | 2 |
| Kleinschmidt-Demasters, 1996 | 58/M | L3 | 2 | 26 | 0 |
| Kleinschmidt-Demasters, 1996 | 60/M | T8-10, L1 | 1 | 1 | 0 |
| Moriyama, 1997 | 64/M | N/A | 1 | 7 | N/A |
| Solau-Gervais, 1998 | 29/M | L2 | N/A | N/A | N/A |
| Frappaz, 1999 | 52/M | L | 1 | 12 | 2 |
| Beauchesne, 2000 | 54/M | T5, 8, 10, 11 | 1 | 9 | 2 |
| Park, 2000 | 31/M | N/A | 1 | 48 | 33 |
| Park, 2000 | 60/M | C1-2, C6 | 1 | 21 | 11 |
| Cervio, 2001 | 44/M | L3-5 | 3 | 17 | 5 |
| Fabi, 2004 | 43/M | L1, 3 | 1 | 84 | 48 |
| Rajagopalan, 2005 | 60/M | L4 | 2 | 20 | 13 |
| Utsuki, 2005 | 42/M | C1, 6 | 2 | 37 | 6 |
| Astner, 2006 | 54/M | N/A | 1 | 36 | 30 |
| Robert, 2008 | 45/F | T7 | 1 | N/A | N/A |
| Pham, 2010 | 61/F | C7 | 1 | N/A | N/A |
| Kalokhe, 2011 | 72/M | N/A | 1 | 19 | 9 |
| Kalokhe, 2011 | 31/F | N/A | 1 | 9 | 5 |
| Blume, 2013 | 40/M | C5, 6 | 3 | N/A | 10 |
| Hamilton, 2014 | 30/F | L5 | 2 | 28 | 17 |
| Kim, 2014 | 39/M | T | 1 | 10 | 3 |
| Khattab, 2015 | 51/M | T | 3 | 46 | 6 |
| Undabeitia, 2015 | 20/F | N/A | 1 | 8 | 3 |
| Starnoni, 2016 | 42/M | C5, 6 | 3 | N/A | N/A |
| Franceschi, 2016 | 70/M | L | 1 | N/A | N/A |
| Xu, 2016 | 58/F | T11, S | 1 | 54 | 25 |
| Simonetti, 2017 | 38/M | T | 1 | 45 | 2 |
| Sun, 2017 | 43/M | N/A | 1 | N/A | 2 |
| Wu, 2017 | 38/F | T, L, S | 2 | 9 | 1 |
| Ricard, 2019 | 37/M | T | 5 | 139 | 2 |
| Li, 2020 | 51/M | C | 1 | 21 | 3 |
| Colamaria, 2021 | 46/F | N/A | 1 | N/A | N/A |
| den Hartog, 2021 | 62/M | N/A | 2 | 45 | 4 |
| den Hartog, 2021 | 59/M | N/A | 1 | N/A | N/A |
| Noch, 2021 | 44/F | T7-8 | 2 | 24.6 | 3.1 |
| Noch, 2021 | 28/M | N/A | 3 | 37.5 | 5 |
| Noch, 2021 | 23/F | L5 | 1 | 37.5 | 5 |
| Zhang, 2021 | 47/M | T7, 10 | 2 | 43 | 12 |
| Present case | 20/M | L1 | 3 | N/A | N/A |

F = female, M = male, N/A = not available

* One of the surgeries in this case includes external decompression.

** Shunt was conducted in this case.
